# Selective Bronchial Occlusion for Treatment of a Bronchopleural Fistula in an Extremely Preterm Infant

**DOI:** 10.3390/children8121208

**Published:** 2021-12-20

**Authors:** Giacomo Simeone Amelio, Mariarosa Colnaghi, Silvia Gulden, Genny Raffaeli, Valeria Cortesi, Ilaria Amodeo, Giacomo Cavallaro, Fabio Mosca, Stefano Ghirardello

**Affiliations:** 1 Neonatal Intensive Care Unit, Fondazione IRCCS Ca’ Granda Ospedale Maggiore Policlinico, 20122 Milan, Italy; giacomo.amelio3@gmail.com (G.S.A.); mariarosa.colnaghi@policlinico.mi.it (M.C.); silvia.gulden@hotmail.it (S.G.); genny.raffaeli@unimi.it (G.R.); valeria.cortesi92@gmail.com (V.C.); amodeoilaria@gmail.com (I.A.); fabio.mosca@unimi.it (F.M.); s.ghirardello@smatteo.pv.it (S.G.); 2 Department of Clinical Sciences and Community Health, Università degli Studi di Milano, 20122 Milan, Italy; 3 Neonatal Intensive Care Unit, Fondazione IRCCS Policlinico San Matteo, 27100 Pavia, Italy

**Keywords:** pneumothorax, selective bronchial occlusion, bronchopleural fistula, air leak, preterm infant

## Abstract

Neonatal pulmonary air leak commonly occurs as a complication of mechanical ventilation in infants with underlying hyaline membrane disease. They can commonly be managed conservatively or with the application of a chest drain, but some severe cases pose a significant challenge in finding an alternative therapeutic solution. Selective bronchial occlusion represents an unconventional rescue therapy for treating bronchopleural fistula resistant to the standard therapy. A 27-week gestation preterm infant ventilated for respiratory distress syndrome developed tension right-sided pneumothorax. Conventional modalities of treatment were tried and were unsuccessful. Intermittent selective bronchial occlusion with a Fogarty’s catheter and high-frequency oscillatory ventilation resulted in considerable improvement in the infant’s clinical condition and radiographic findings.

## 1. Introduction

Pulmonary air leak is a possible complication in preterm infants receiving mechanical ventilation for respiratory distress syndrome [1]. It is caused by an over-stretching and subsequent rupture of the alveolar wall leading to an aberrant communication between the alveolar space and a different anatomic site. The main forms are pulmonary interstitial emphysema (PIE), pneumomediastinum, pneumopericardium, and pneumothorax [2]. However, the appropriate management of this condition is not always clearly defined, and various therapeutic strategies have been reported in case of failure of conventional therapy [3].

We report our experience with an extremely preterm infant affected by persistent right bronchopleural fistula treated successfully with selective bronchial occlusion and high-frequency oscillatory ventilation (HFOV).

## 2. Case Presentation

**Figure 1:**
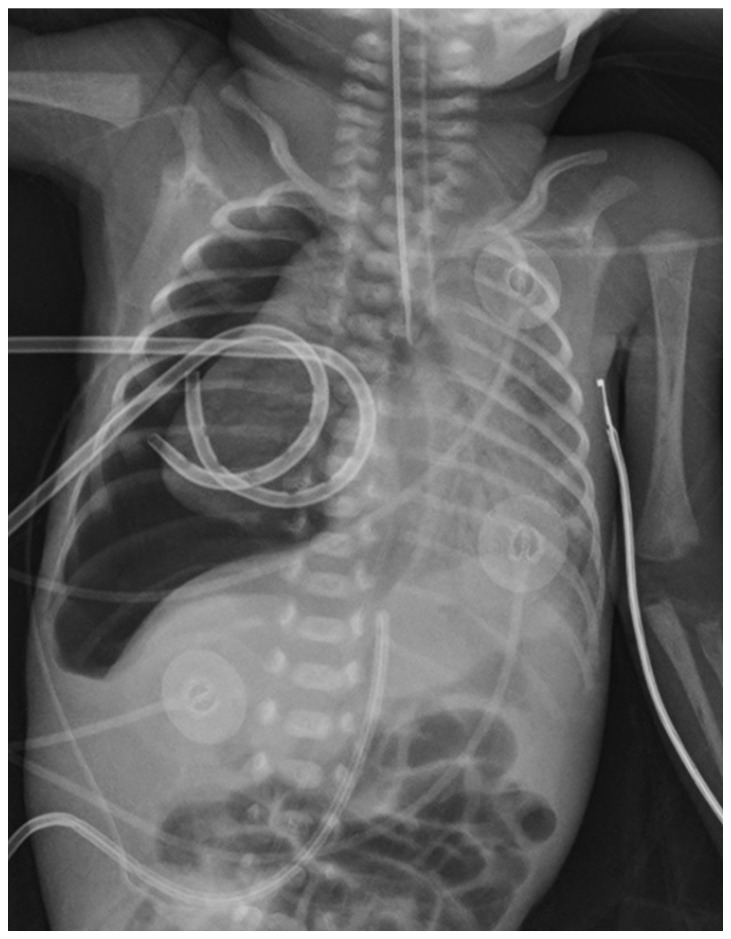
X-ray on the first day of life. Tension right-sided pneumothorax with the leftward shift of the mediastinum after surfactant administration.

Parents provided written informed consent for the publication of this case report, including images. All information revealing the subject’s identity was avoided, and all information was anonymized. A 600 g preterm male was born at 27 weeks gestation by cesarean section due to intrauterine growth reduction associated with pathological placental flows. The APGAR score was 4 and 8 at 1 and 5 min, respectively. At 4 h, poractant alfa (Curosurf, Chiesi Pharmaceuticals, Parma, Italy) was administered by the less invasive surfactant administration (LISA) technique for a hyaline membrane disease, but a tension right-sided pneumothorax occurred almost immediately (Figure 1).

Stabilization proved difficult with a consistently high flow through the bronchopleural fistula requiring the application of two suction drains at −10 cmH_2_O. Simultaneously, mechanical ventilation was started (PIP 25, MAP 10, FiO_2_ 0.4) and the infant was positioned in right lateral decubitus.

**Figure 2:**
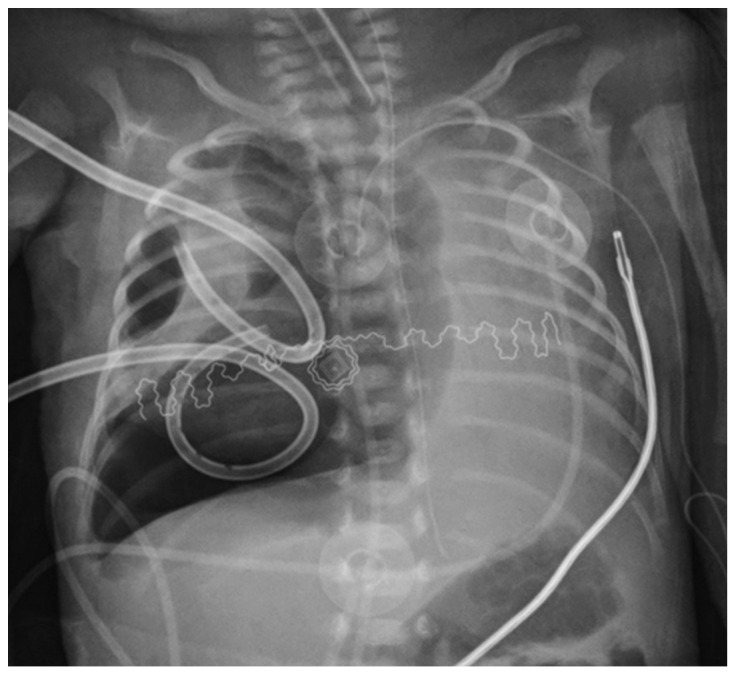
X-ray in 16th day of life. Recurrence of pneumothorax despite two suction chest drains.

Despite postural therapy and the maintenance of two suction drains, the respiratory condition remained critical in the following days. Ultrasound and X-ray checks repeatedly documented the reduction of the pneumothorax size. Still, the two drains showed continuous bubbling in the water seal chamber, meaning a persistent bronchopleural leakage. Furthermore, during the patient’s daily care, the drains presented transient obstructions due to their tiny size, leading to the immediate recurrence of the right pneumothorax and clinical destabilization. On day of life (DOL) 16, the infant deteriorated, and radiographs indicated a worsening pneumothorax associated with further hypoxemia (Figure 2).

**Figure 3:**
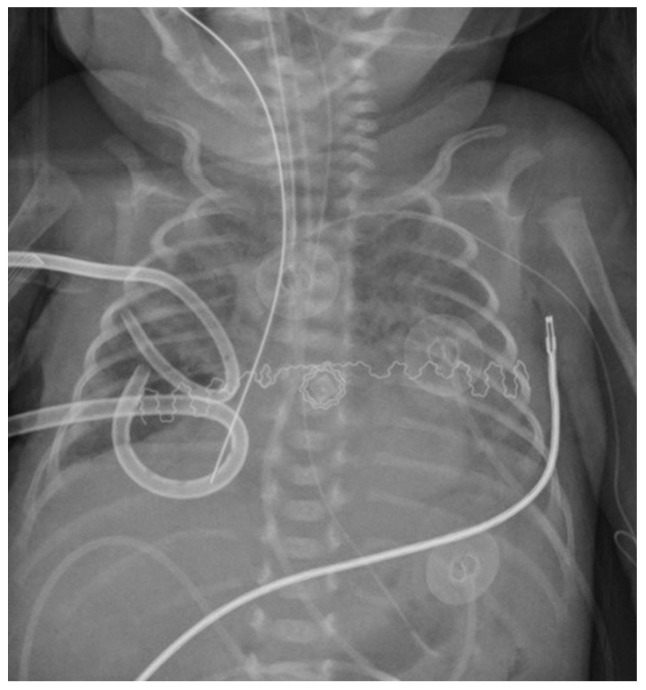
X-ray on the 17th day of life, 24 h after the occlusion. The Fogarty’s catheter in the right bronchus reduces pneumothorax and partial collapse of the right lower lobe.

After obtaining informed parental consent, a 3 French Fogarty’s catheter was entered through the vocal cords to protect the right lung from further injury. The tip was placed in the right main bronchus before intubation with a new endotracheal tube. The catheter was inserted 11 cm from the lips by direct laryngoscopy, approximately 4 cm deeper than the conventional orotracheal tube position. The insertion depth was previously calculated by adding the length of the endotracheal tube, the distance measured from its tip, and the desired position. The Fogarty catheter was spontaneously directed in the right main bronchus using the anatomical alignment with the trachea compared to the left main bronchus [4]. A chest X-Ray confirmed its exact position, and the 5 mm long balloon, located 1 cm from the tip, was inflated with 0.2 mL of air. The flow through the thoracic drains immediately stopped, and HFOV was started (MAP 14 cmH_2_O, Delta P 27 mmHg, Hz 14, FiO_2_ 1.0). The balloon was deflated for 2 h for every hour inflated to minimize bronchial wall pressure necrosis. The patient tolerated the procedure well. Hypoxemia was rapidly corrected, with FiO_2_ decreasing up to 0.4 in forty-eight hours. On DOL 17, a chest x-ray showed a substantial reduction of pneumothorax associated with the partial exclusion of the right lower lobe from ventilation and gradual reaeration of the left lung, and then suction was stopped (Figure 3).

On DOL 19, the Fogarty catheter was removed, conventional ventilation was reestablished, and the drains were clamped intermittently and removed on DOL 21. Finally, on DOL 23, the patient was extubated and discharged home at 39 weeks of postmenstrual age in typical room air.

## 3. Discussion

Pneumothorax represents the most frequent form of pulmonary air leak in preterm infants, with an incidence ranging from 3 to 9% in very low birth weight infants, and is associated with increased mortality and morbidity [3]. Conventional treatment of symptomatic pneumothorax consists of the application of a chest drain and downward lateral positioning of the affected hemithorax associated with low tidal volume ventilation, more easily obtained with HFOV (1.5–2.5 mL/kg) than conventional mechanical ventilation (4–6 mL/kg) [2,4,5]. Selective bronchial intubation of the nonaffected lung and selective bronchial occlusion of the affected lung are two additional treatment options described more often in other forms of neonatal air leak such as PIE, whose evidence is based on single case reports, and small case series [1]. However, the first seems to have a low success rate and an increased risk of complications such as hypoxia associated with reintubation and acquired bronchial stenosis [6,7]. In turn, successful selective bronchial occlusion has been reported in less than 30 cases of premature infants with different occlusion techniques [4,8,9,10,11,12,13,14,15,16]. Characteristics of these patients and the procedure used are summarized in Table 1.

In our case, suction chest drains and postural therapy were immediately applied, but the multiple relapses of pneumothorax associated with periods of hypoxemia exposed the infant to prolonged artificial ventilation at high concentrations of oxygen. This condition represented a challenge in the ventilation strategy due to a self-perpetuated process, which required a further increase in ventilation pressure, which led to greater air escape through the bronchopleural fistula. The situation made it necessary to partially exclude the bronchopleural fistula from ventilation and reduce the tidal volume through the HFOV.

The literature review of our previous experience prompted us to perform selective bronchial occlusion [4]. The catheter was easily placed through the trachea in the right bronchus; the left side may be more difficult. However, the procedure proved safe, no adverse side effects were noted, and there was no recurrence of pneumothorax. In addition, the inflation of the balloon caused a rapid clinical and radiologic reduction of the air in the pleural cavity.

## 4. Conclusions

In conclusion, selective bronchial occlusion with Fogarty’s catheter insertion remains a rescue therapy. However, it may be important for neonatologists to be aware of this procedure in cases of persistent bronchopleural fistula. In addition, the technical appropriateness of the instrumentation used, the best length of stay of the catheter, and the best strategy for intermittent deflation and inflation of the balloon remain to be explored in order to improve its effectiveness and minimize possible complications.

## Figures and Tables

**Table 1:** Overview of successful selective bronchial occlusion cases reported in the literature in preterm infants.

Author	GA (Weeks)	Weight (g)	Air Leak	Position	Unsuccessful Treatment	HFO	Instrumentation Used	Insertion	Occlusion Type	Length
Present study	27	600	PTX	Right	Chest drainage and postural therapy	Yes	Fogarty’s catheter 3 FR, inflated 0.2 mL	Direct laryngoscopy	Inflated 1 h/deflated 2 h	72 h
Al alaiyan	30	1500	PIE	Right	Postural therapy and chest drainage	Yes	Balloon catheter inflated 0.3 mL	Direct laryngoscopy	Deflated for 5 min each hour	48 h
Alijishi	28	1240	PIE	Right	Postural therapy, chest drainage, and selective left bronchial intubation	No	Balloon catheter inflated 0.35 mL	Direct laryngoscopy	Deflated for 5 min each hour	60 h
Auerbach	30	1620	PIE	Left	Chest drainage and selective right bronchial intubation	No	Fogarty’s catheter 4 FR	Bronchoscopy	Continuous	48 h
	32	1250	PIE	Left	NA	No	Fogarty’s catheter 3 FR	Bronchoscopy	Inflated 4 h/deflated 4 h	96 h
Dewitte	29	1340	PIE	Right	Postural therapy	NA	ET 2.5 mm modified *	Direct laryngoscopy	Continuous	72 h
	28	1120	PIE	Right	Chest drainage	NA	ET 2.5 mm modified *	Direct laryngoscopy	Continuous	48 h
Hathorn	33	2390	PIE	Right	NA	NA	Balloon catheter 4 FR	Direct laryngoscopy	Continuous	96 h
	27	1200	PIE	Left	NA	NA	Balloon catheter 4 FR	Bronchoscopy with Fluoroscopic guidance	Continuous	7 days
Lewis	26	760	PIE	Right	Postural therapy, chest drainage, and selective left bronchial intubation	No	Swan-Ganz catheter 5 FR	Direct laryngoscopy	Deflated 5 min each hour	72 h
Mathew	29	950	PIE and PTX	Right	Chest drainage and selective left bronchial intubation	No	Umbilical catheter modified °, inflated 0.7 mL	Direct laryngoscopy	Continuous	48 + 72 h Ψ
Mosca	26	1000	PIE and PTX	Right	Postural therapy and chest drainage	No	Fogarty’s catheter 5 FR, inflated 0.3 mL	Direct laryngoscopy	Deflated 5 min each hour	26 h
Weintraub	28	1200	PIE	Right	Postural therapy	No	Balloon catheter 4 FR, inflated 0.75 mL	Direct laryngoscopy	Continuous	NA
Rastoigi ⊥	25–32	600–1700	12 PIE/2 PTX	NA	Postural therapy	3 yes/11 no	Swan-Ganz catheter 5 FR, inflated 0.6 mL	Direct laryngoscopy	Every hour at the hour	24 h after the PIE resolution

PIE = pulmonary interstitial emphysema; PTX = pneumothorax; ET = endotracheal tube; NA = not available; FR = French (1 Fr = 1/3 of mm). * An adapted ET positioned with the apex in the right bronchus, with the distal opening occluded and a hole along the radiopaque line starting at 1 cm and ending 2 cm from the distal end. ° An adapted umbilical catheter attached to a handmade latex esophageal pressure balloon, length 1.25 cm, width 1 cm. Ψ The first occlusion lasted 48 h, followed by a second occlusion of 72 h for a recurrence of PIE. ⊥ A case series reporting 14 cases of successful selective bronchial occlusion in premature babies, the history of each infant is not available. Gestational age and weight ranges are reported.

## Data Availability

The data supporting this study’s findings are not publicly available because they contain information that could compromise participants’ privacy, but are available from the corresponding author.
